# Workplace violence in a 20 year follow-up study of norwegian physicians: The roles of gender, personality and stage of career

**DOI:** 10.1192/j.eurpsy.2021.986

**Published:** 2021-08-13

**Authors:** S. Nøland, H. Taipale, J. Mahmood, R. Tyssen

**Affiliations:** 1 Faculty Of Medicine, University of Oslo, Oslo, Norway; 2 Department Of Behavioural Medicine, University of Oslo, Oslo, Norway

**Keywords:** workplace violence, threats, Predictors, longitudinal study

## Abstract

**Introduction:**

Workplace violence (WPV) is a worldwide health problem with major individual and societal consequences. Previously identified predictors of WPV include working in psychiatry and work stress.

**Objectives:**

To investigate WPV trends during Norwegian doctors’ careers and assess individual long-term predictors in a longitudinal study.

**Methods:**

Two nationwide medical student cohorts (n=1052) who graduated 6 years apart were surveyed at graduation (T1, 1993/94 and 1999) and 4 (T2), 10 (T3), 15 (T4) and 20 (T5) years after graduation (Figure 1). WPV was measured as multiple threats or acts of violence experienced at least twice. Individual predictors were obtained at T1 and work-related factors at T2–T5. WPV was analysed using repeated measures (Generalized Estimating Equations).

**Results:**

The prevalence of multiple threats and acts of violence declined at T2–T5 (p<0.001). Adjusted predictors of threats were male gender (odds ratio, OR 2.76, [95% confidence interval] 1.73–4.40; p<0.001), vulnerability traits (OR 0.90, [0.82–0.99]; p=0.031), older cohort (OR 1.63,[1.04–2.58], p=0.035) and working in psychiatry (OR 7.50, [4.42–12.71]; p<0.001). Adjusted predictors of acts were male gender (OR 3.37, [1.45–7.84]; p=0.005), older cohort (OR 6.08, [1.68–21.97]; p=0.006) and working in psychiatry (OR 12.34, [5.40–28.23]; p<0.001).

**Conclusions:**

Higher rates of multiple threats and acts of violence were observed during early medical careers, with men at higher risk. Low levels of vulnerability traits (neuroticism) predicted independently the experience of violent threats. A cohort effect indicated a reduction in WPV (both threats and acts) in the younger cohort.

